# Blueberry-on-Top Phenomenon in Apical Variant Hypertrophic Cardiomyopathy

**DOI:** 10.1016/j.case.2024.01.006

**Published:** 2024-03-03

**Authors:** Saed Alnaimat, Mariah Mascara, Georgios Lygouris, Robert W.W. Biederman

**Affiliations:** a Department of Cardiology, Allegheny General Hospital, Pittsburgh, Pennsylvania; b Department of Internal Medicine, Allegheny General Hospital, Pittsburgh, Pennsylvania; c West Virginia University School of Medicine, Morgantown, West Virginia; d Bioengineering Department, Carnegie Mellon, University, Pittsburgh, Pennsylvania; e Medical University of South Carolina and Roper/SF Hospital, Charleston, South Carolina

**Keywords:** Apical hypertrophic cardiomyopathy, Echocardiography, Strain imaging, Speckle-tracking

## Abstract

• “Inverse-amyloid” or “blueberry-on-top” strain pattern is a finding in AHCM.
• The time to peak strain parametric map is the best depiction of this unique pattern.
• This pattern provides a supportive diagnostic feature of apical variant HCM.

## Graphical abstract

**Figure:**
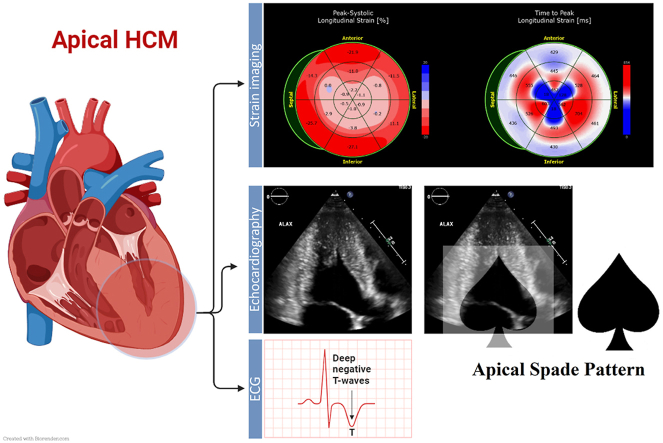

## Introduction

Apical hypertrophic cardiomyopathy (AHCM) is a rare phenotypic variant of hypertrophic cardiomyopathy (HCM), most commonly seen in Asian men (Yamaguchi syndrome). Apical HCM is characterized by hypertrophy predominantly affecting the cardiac apex, with an “ace of spades”–shaped left ventricular (LV) cavity best seen on the 4-chamber view of a transthoracic echocardiogram (TTE). However, TTE can be falsely negative in 30% of AHCM cases, largely due to difficulties in delineating endocardial border.^1^^,^^2^ Herein, we introduce the “blueberry-on-top” phenomenon, a characteristic myocardial strain finding on TTE, to help identify AHCM cases.

## Case Presentation

We present an 83-year-old woman with well-controlled hypertension, paroxysmal atrial fibrillation (AF), high-grade atrioventricular block post–biventricular pacemaker (BiV-PPM), and chronic obstructive pulmonary disease who presented with progressive dyspnea on exertion and fatigue for several months, resulting in New York Heart Association functional class II symptoms. The patient denied chest pain, palpitations, syncope, orthopnea, or lower-extremity edema. Vital signs were remarkable for a blood pressure of 128/82 mm Hg, heart rate of 78 beats per minute, respiratory rate of 16 breaths per minute, and oxygen saturation of 96% on room air. The physical examination showed clear lungs to auscultation. Cardiac auscultation was notable for irregularly irregular rhythm, normal S1 and S2, and no murmur. There was no jugular venous distention but a prominent V wave consistent with tricuspid regurgitation (TR). Peripheral pulses were normal, and no lower-extremity edema was noted.

**Figure 1:**
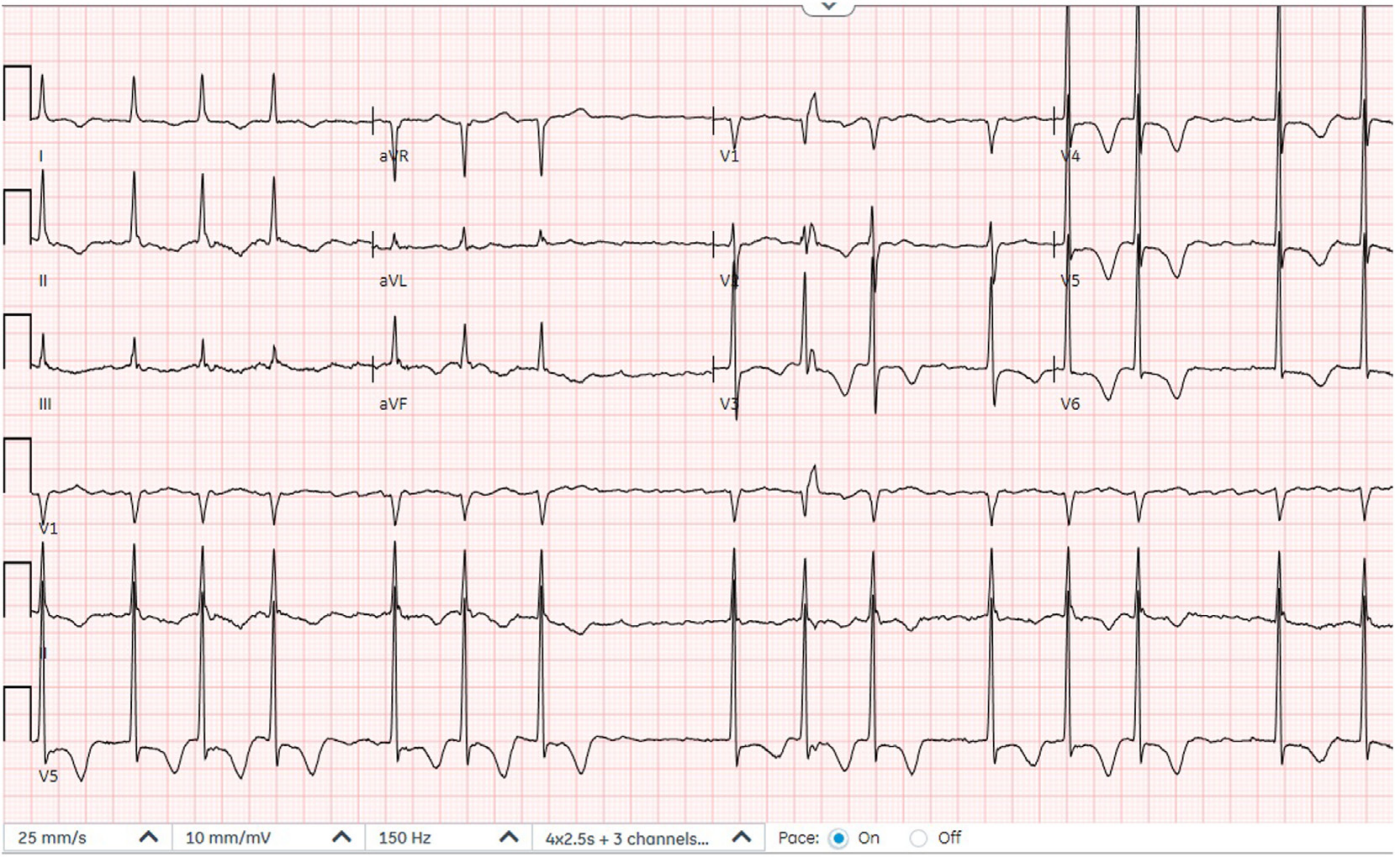
Electrocardiogram demonstrates AF with deep negative T waves in the precordial leads.

The patient initially underwent a pulmonary function test that showed a mild obstructive and restrictive lung disease. Subsequently, the patient had cardiac evaluation including an electrocardiogram (ECG) that showed AF with LV hypertrophy (LVH) and symmetrical deep negative T waves in the precordial leads (Figure 1).

**Figure 2:**
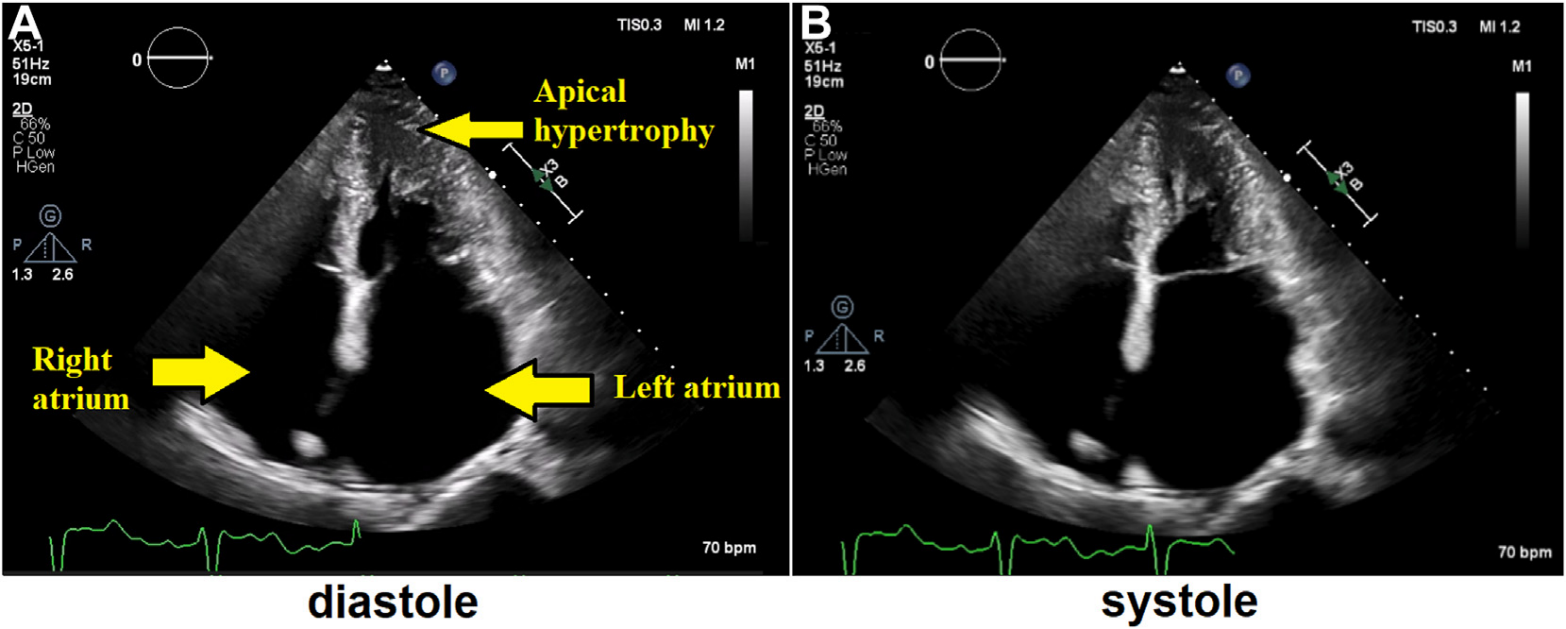
Two-dimensional TTE, apical 4-chamber view in diastole **(A)** and systole **(B)**, demonstrates progressive mid-to-apical LV wall thickness and severe biatrial dilation.

**Figure 3:**
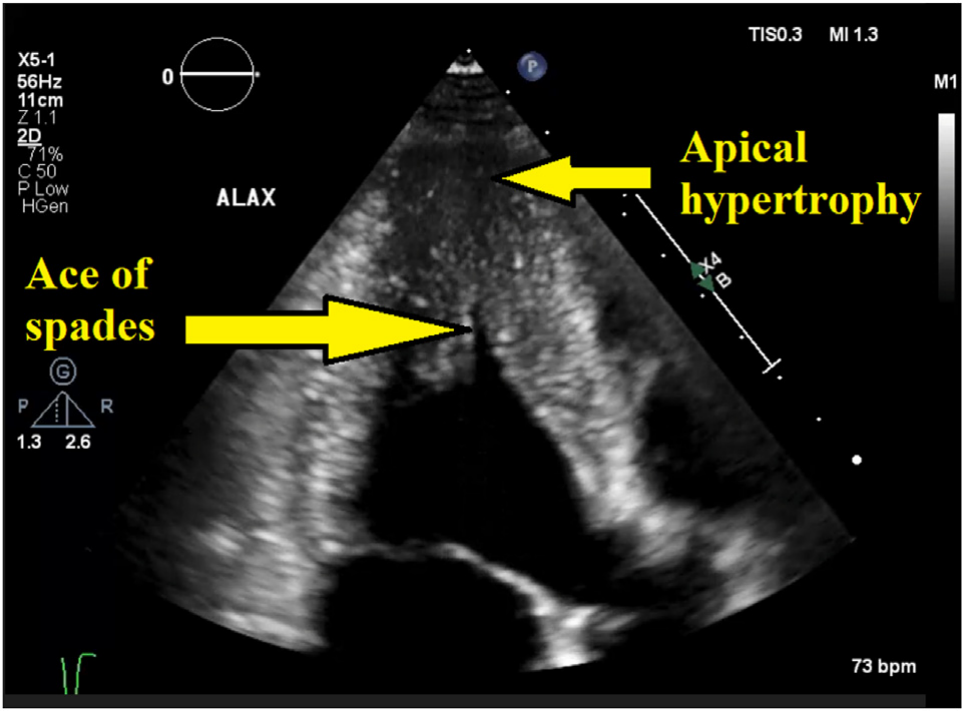
Two-dimensional TTE, apical 3-chamber view, diastolic phase, demonstrates LV apical hypertrophy with an ace of spades–shaped LV cavity.

**Figure 4:**
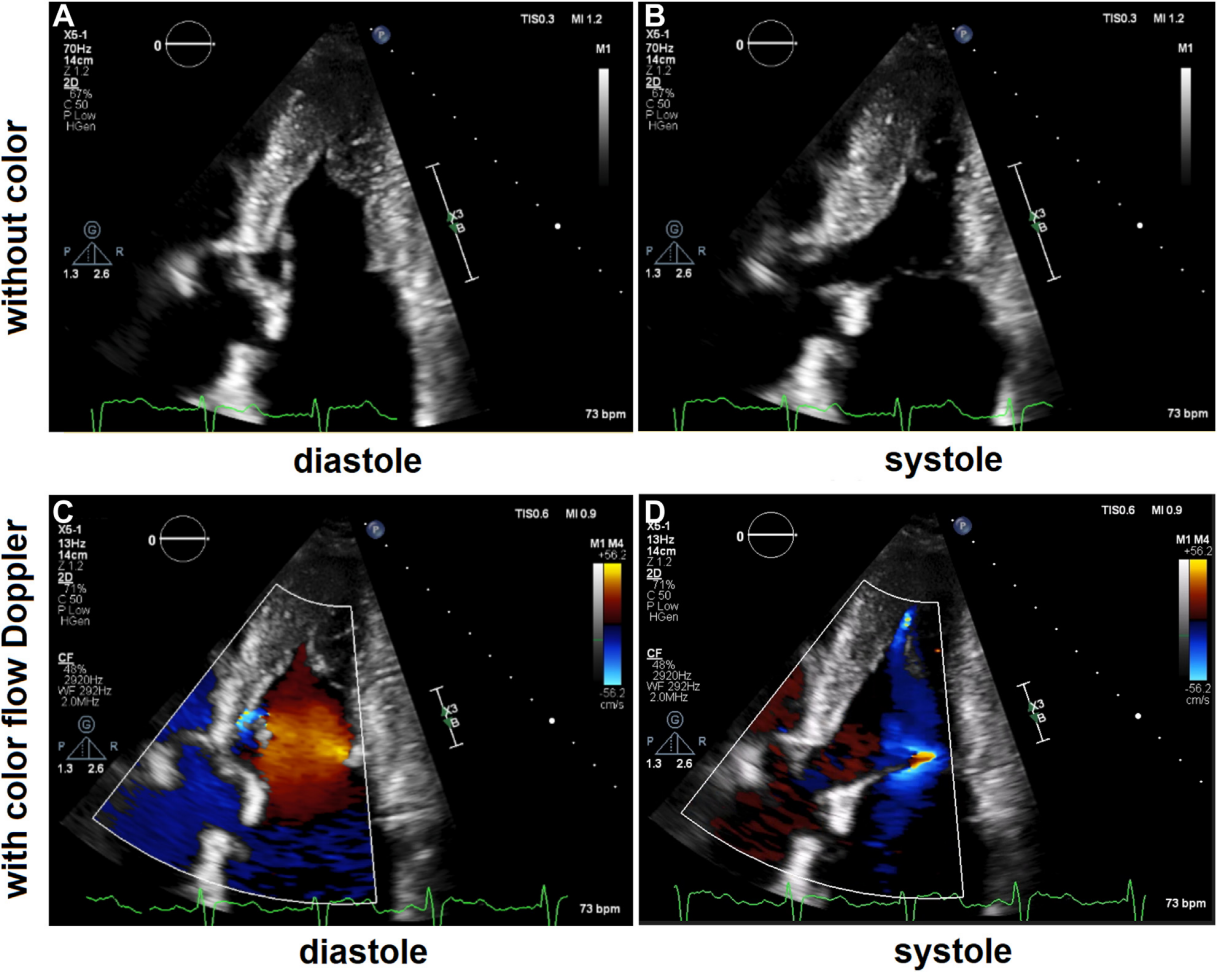
Two-dimensional TTE, apical 5-chamber view without (*top*) and with (*bottom*) color-flow Doppler in diastole (*left*) and systole (*right*), demonstrates AHCM with a slit-like LV cavity toward the cardiac apex.

Device interrogation showed normal device function with 99% biventricular pacing and no ventricular arrythmias. While image quality was suboptimal, TTE demonstrated a progressive mid-to-apical LVH (20 mm) with an ace of spades–shaped LV cavity and severe biatrial enlargement (left atrial volume index, 105 mL/m^2^; right atrial volume index, 76 mL/m^2^). Left ventricular function was hyperdynamic (LV ejection fraction [LVEF] was visually estimated >75%), but biplane quantitation was obfuscated by extensive LVH pattern. Given the underlying AF and biventricular pacing, it was not possible to accurately grade diastolic dysfunction. Mitral E-velocity deceleration time was 177 ms, and the E to annular e' velocity ratio (E/e' ratio) was 11.3.^3^ The right ventricle was normal in size with mildly reduced systolic function (fractional area change of 34%). No systolic anterior motion of mitral valve (SAM) or elevated LV outflow tract (LVOT) gradients were noted. There was mild mitral regurgitation, severe TR, and moderate pulmonic regurgitation. Right ventricular systolic pressure was estimated at 29 mm Hg (although it could be underestimated in the presence of severe TR). A moderate posteriorly located pericardial effusion was noted as well (Figure 2, Figure 3, Figure 4, Videos 1-3).

**Figure 5:**
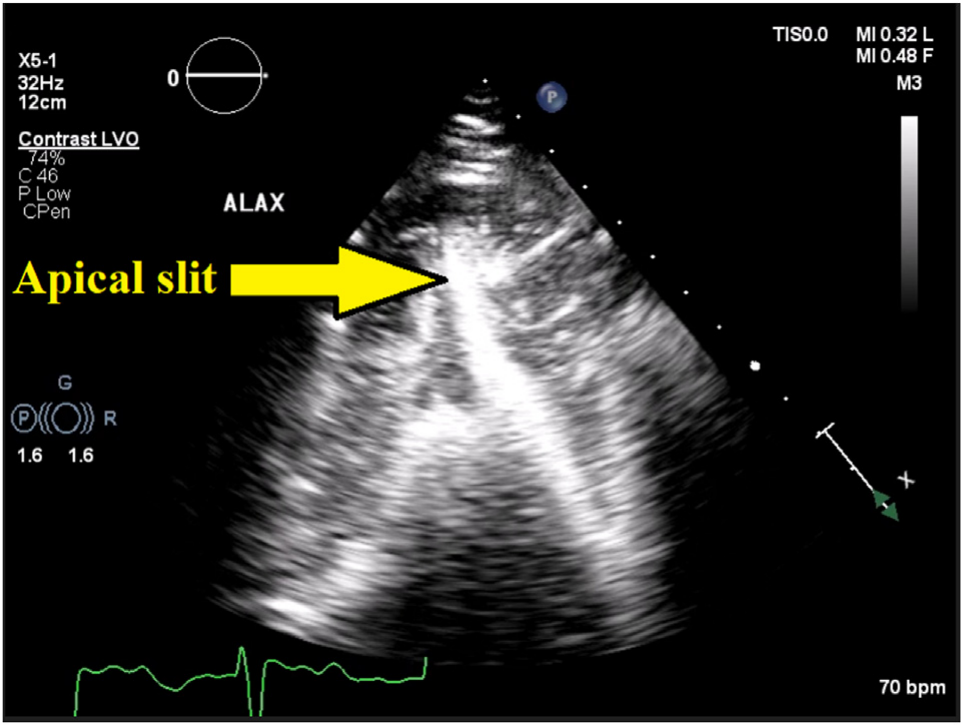
Two-dimensional TTE, apical long-axis view in diastole after administration of an ultrasound-enhancing agent, demonstrates the slit-like LV cavity extending into the apical tip.

Contrast-enhanced echocardiography (using activated perflutren lipid microspheres) showed an ace of spades–shaped LV cavity extending into the slit-like apical tip without aneurysm formation (Figure 5, Videos 5 and 6). Although some retained contrast was noted in the very apex in the apical 2-chamber view (Video 5), no apical aneurysm was seen on the rest of the views including contrast-enhanced apical 4-chamber view (Video 6).

**Figure 6:**
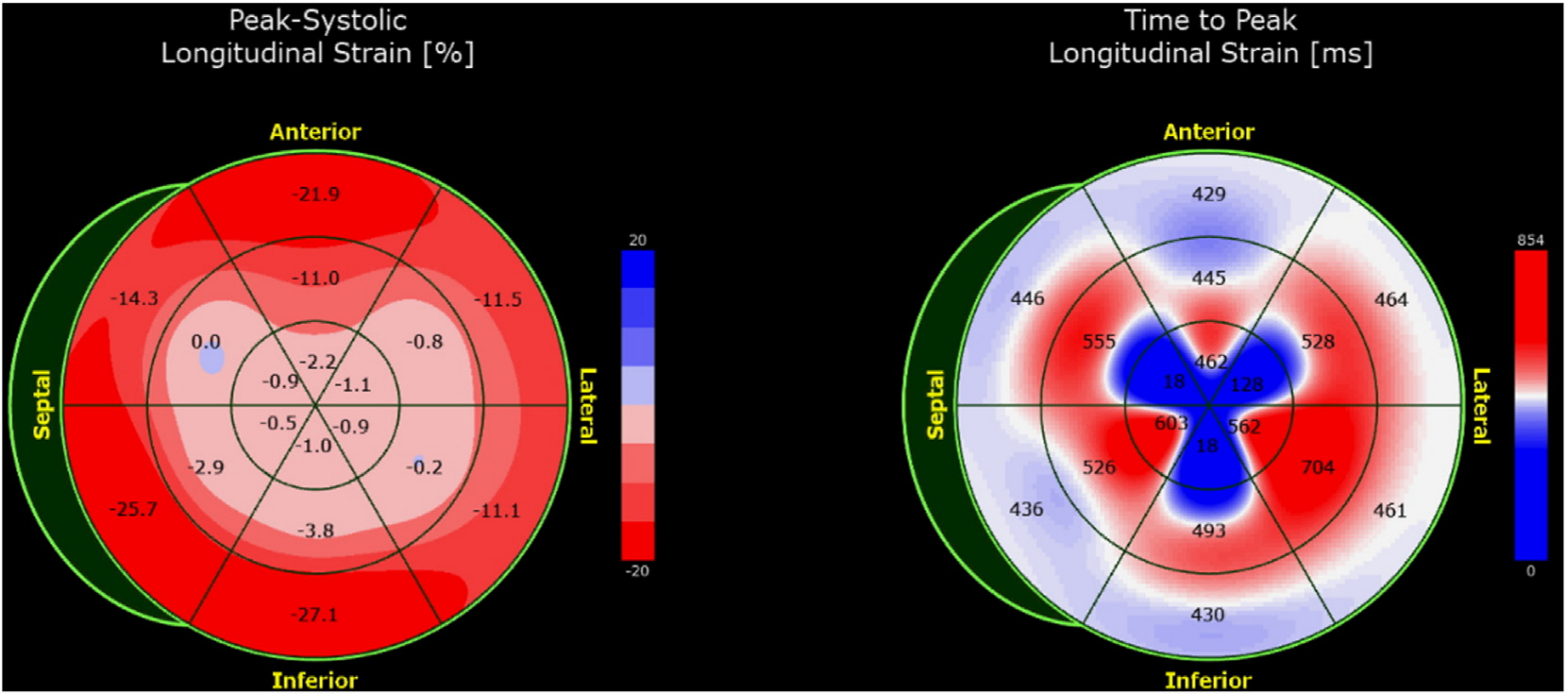
Parametric maps (bull’s-eye displays) of myocardial global longitudinal peak systolic strain (*left*) and time to peak longitudinal strain (*right*) demonstrate the characteristic blueberry-on-top patterns.

Interestingly, while peak longitudinal strain values were modestly reduced at the apex, there was a paradoxical markedly advanced time to peak strain, demonstrating a blueberry-on-top phenomenon (Figure 6).

**Figure 7:**
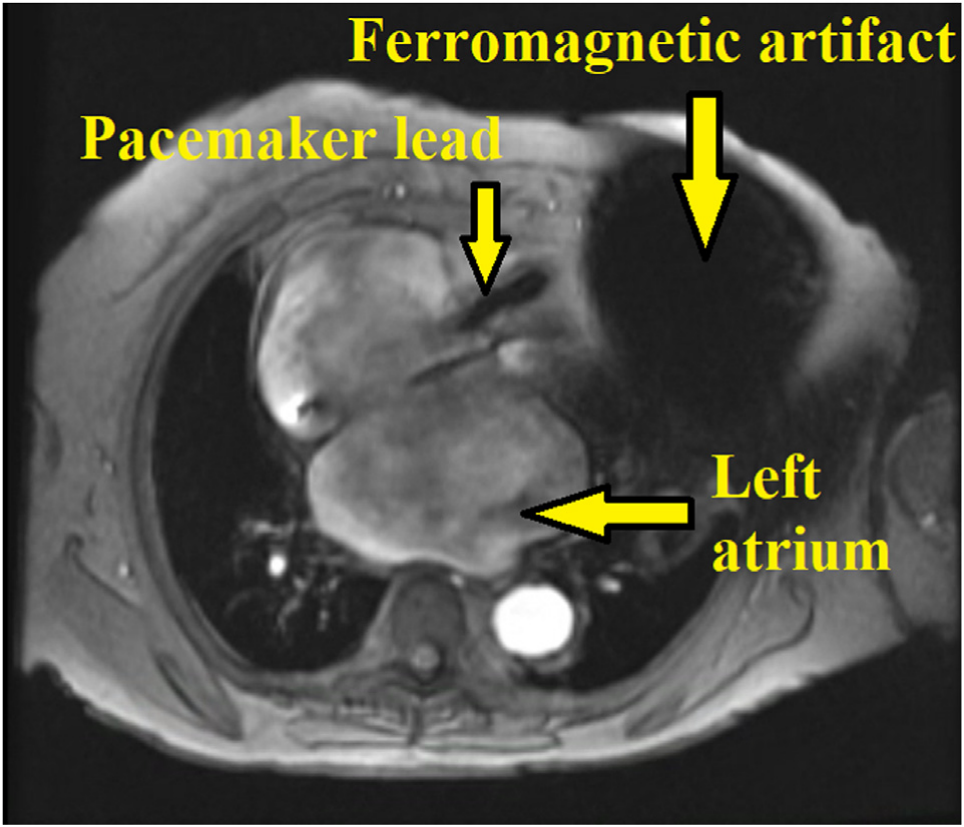
Cardiac magnetic resonance imaging (1.5 T scanner), cine spoiled gradient echo sequence, axial 4-chamber view, demonstrates the presence of severe ferromagnetic artifacts (*vertical arrows*) from biventricular pacemaker generator and leads that obscures visualization of the LV myocardium.

Cardiovascular magnetic resonance (CMR) was attempted but was nondiagnostic due to severe ferromagnetic artifacts from BiV-PPM (Figure 7). Given the new diagnosis of AHCM, the patient and their family were offered genetic testing, which showed positive results for SOS1, ALMS1, DSP, and SCN5A mutations in the patient (variants of uncertain significance), while the granddaughter was positive for the MYH7 pathogenic variant. The patient was started on guideline-directed medical therapy for diastolic heart failure with furosemide 40 mg daily and empagliflozin 10 mg oral daily, and was continued on metoprolol tartrate 50 mg twice daily and apixaban 2.5 mg twice daily. The patient was seen in the advanced heart failure clinic a few months later and had moderate improvement in symptoms since starting diuresis. The patient was counseled on maintaining euvolemia and participating in a daily, light aerobic activity exercise regimen. Risk stratification for sudden cardiac death (SCD) showed a low-risk profile and no indication for an implantable cardioverter-defibrillator (ICD) implantation/upgrade.^4^ Severe TR was managed medically without the need for surgical or transcatheter intervention.

## Discussion

Hypertrophic cardiomyopathy is an umbrella term for heterogeneous heart muscle diseases characterized by LVH not attributable to abnormal cardiac loading conditions. Long after establishing its morphological definition, the genetic basis of HCM was discovered in the early 1990s, and it is now known that HCM is predominantly caused by autosomal dominant mutations in sarcomeric protein genes.^1^ Approximately 30% to 60% of patients with HCM have an identifiable pathogenic or likely pathogenic genetic variant. Yet a substantial proportion of patients with HCM have no evidence of a genetic etiology of their disease and have no other affected family members. Hypertrophic cardiomyopathy is a genetic heart disease with highly evident and devastating complications, and it has been regarded as one of the most common causes of SCD among young adults in North America.^4^

Several morphologic variants of HCM have been identified, including asymmetric basal septal (or “classic” HCM), concentric, reverse septal, neutral, and apical (AHCM). Apical HCM is a rare form of HCM that was first introduced by Sakamoto *et al.*^5^ in 1976 in Japanese patients who had localized hypertrophy near the LV apex associated with giant negative T waves on ECG. In 1979, Yamaguchi *et al*.^6^ were the first to characterize the syndrome and its ventriculographic features including a “spade-like” configuration of LV cavity in end diastole (Yamaguchi syndrome). The prevalence of HCM ranges from 1:200 to 1:500 in the United States, while AHCM accounts for up to 25% of HCM in Asian populations and 1% to 10% in non-Asians. Apical HCM is more prevalent in men than women, with ratios typically 1.6:1 to 2.8:1. The average age at presentation is 41.4 ± 14.5 years. Apical HCM was originally considered a benign entity, although recent data suggest associated pathology with left atrial dilatation, increased LV filling pressures, elevated blood cardiac protein biomarkers, apical aneurysm, and myocardial scar formation. Further, pathophysiology of AHCM involves small-vessel disease resulting in myocardial ischemia from cavity obliteration and the persistence of apical contraction into early mid-diastole. This can result in microvascular obstruction in the apical segments, regional myocardial perfusion defects, and chest pain.^1^ Other clinical manifestations of AHCM include palpitation, dyspnea, fatigue, syncope, SCD, malignant arrhythmias, and apical infarction with apical aneurysm formation. Yet cardiovascular mortality is generally lower than in other variants of HCM.

Diagnosis of HCM is usually triggered by the identification of a family history of HCM, symptoms such as syncope or cardiac event, a heart murmur during physical examination, abnormal echocardiography obtained for other indications, or an abnormal 12-lead ECG. Hypertrophic cardiomyopathy is mainly an imaging diagnosis since symptoms and ECG changes are not specific for this disorder. According to the 2020 American Heart Association/American College of Cardiology guidelines,^4^ diagnostic criteria for HCM in adults is based on a maximal end-diastolic wall thickness of ≥15 mm anywhere in the left ventricle, in the absence of another cardiac, systemic, or metabolic disease capable of producing such a magnitude of hypertrophy and for which a disease-causing sarcomere (or sarcomere-related) variant is identified or genetic etiology remains unresolved. More limited hypertrophy (13-14 mm) can be diagnostic when present in family members of a patient with HCM or in conjunction with a positive genetic test. For children, the diagnostic criteria are confounded by needing to adjust for body size and growth. A number of other morphologic abnormalities that can be part of the phenotypic expression of the disease (ancillary features) include hypertrophied and apically displaced papillary muscles, aberrant or anomalous insertion of the papillary muscle directly in the anterior leaflet of the mitral valve (in the absence of chordae tendinae), elongated mitral valve leaflets, myocardial crypts, myocardial bridging, and right ventricular hypertrophy.

Echocardiography plays an integral role in the initial screening as well as monitoring of patients with HCM. It can differentiate between different morphologic phenotypes of HCM and reveal apical hypertrophy. Additionally, abnormalities of papillary muscles and mitral valve (including SAM), resting or provocable LVOT obstruction and gradients, and other prognostic features such as diastolic dysfunction, midventricular obstruction and cavity obliteration, or apical aneurysms can be characterized with echocardiography. Doppler assessment of diastolic gradient between the apex and LV cavity also plays an important prognostic role. It was found to increase thromboembolic risk, ventricular arrhythmias, and perfusion abnormalities.^2^ While echocardiography provides valuable diagnostic and prognostic information in suspected and confirmed AHCM, echocardiographic technology has its known limitations and pitfalls. Echocardiography is dependent on the operator, patient anatomy, and reader interpretation. Some patients may not have good acoustic windows to permit visualization of all myocardial segments. A common problem with two-dimensional echocardiogram is foreshortening of the LV cavity, which not only interferes with accurate volumetric analysis but can lead to improper evaluation of LV apex such as missing an apical aneurysm. The left ventricle should taper to an ellipsoid shape at the apex. If the ventricle is foreshortened, the apex has a more rounded appearance.^7^ Such apical foreshortening can also interfere with proper LV strain assessment, which may translate into poor tracking and nondiagnostic LV strain plots. Therefore, it is important to obtain multiple orthogonal views when evaluating LV anatomy to avoid missing such apical aneurysms or dyskinesia. Advanced cardiac imaging techniques such as CMR or cardiac computed tomography do not have this foreshortening limitation and may provide more definitive and reproducible information in some patients.

Imaging the apex remains a potential technical challenge, particularly in the presence of massive hypertrophy and systolic cavity obliteration, which prevents the creation of an ultrasound echo from the endocardium due to lack of acoustic mismatch. In fact, lack of endocardial visualization can sometimes leave the “expanding” epicardial border as the main image creator, leading to a misdiagnosis of apical dyskinetic aneurysm. Such “acoustic mismatch” can be countered by using ultrasound-enhancing agents, which add significant diagnostic information in these patients and aid in proper visualization of endocardial borders. Other options for sonographers to improve near-field imaging include moving the transducer beam focus to the apex, use of single focus rather than multiple focal zones, optimizing dynamic range, and use of narrower widths to yield better lateral resolution. With the availability of broad-bandwidth transducers, it is now relatively easy to modify the transmit frequency. The typical range of frequencies used in adult echocardiography is 2.0 to 5.0 MHz, with higher frequencies producing better image resolution of the cardiac apex. It is recommended that sonographers start with the highest possible frequency throughout the examination then adjust to lower frequencies if additional penetration of the sound wave is needed.^7^ Three-dimensional (3D) echocardiography may offer more insight into the mechanics of SAM and deformational geometry of the LVOT and provide more accurate assessment of the LVOT area after septal reduction therapies. Three-dimensional echocardiography-derived estimations of LVEF and LV mass in hypertrophied hearts compare more favorably with CMR imaging.^8^

Apical aneurysms occur in 2% of patients with HCM and 13% to 15% with AHCM and confer risk of apical thrombus formation and thromboembolic stroke. Apical aneurysms have been associated with SCD, monomorphic ventricular tachycardia, LV systolic dysfunction, and heart failure. A clue to their presence is the persistence of apical blood pooling distal to the point of apical systolic cavity obliteration. Small apical aneurysms are often overlooked on two-dimensional echocardiography and require an ultrasound-enhancing agent or advanced cardiac imaging for detection. These are associated with higher thromboembolic and adverse event rates when compared to aneurysms associated with midcavity HCM.^1^

A few phenotypic “mimickers” of AHCM have been described in the literature, including Fabry’s disease and athlete’s heart. It has been reported that up to 23% of patients with Fabry’s disease with LVH have an AHCM pattern by CMR, while 2% of highly trained male athletes may have “gray zone” LV wall thickness of 13 to 15 mm. Interestingly, highly trained female athletes rarely show LVH of >11 mm, suggesting that athletic females within the gray zone LVH are more likely to have HCM. Native T1 and extracellular volume values using CMR are lower in athletes than in patients with HCM, and this may serve as an important diagnostic tool in differentiating the 2 conditions. Additionally, ECG abnormalities are more common in true AHCM than in athletes with pure apical LVH.^1^

The cherry-on-top strain pattern is classically seen in patients with cardiac amyloidosis due to amyloid deposition in the basal segments with relative apical sparing. In contrast, blueberry on top is observed in AHCM as the strain is spared in the LV base while the apex is distorted due to myocyte fiber disarray, increased loose connective tissue, and fibrosis, which are thought to interfere with force generation.^9^ While the “inverse-amyloid” peak systolic strain pattern has been previously observed in patients with AHCM,^1^, ^10^, ^11^ time to peak strain seems to be a more robust tool in depicting the characteristic blueberry-on-top phenotype in patients with AHCM. In other words, we recognize that the blueberry-on-top pattern can be manifested on both peak systolic and time to peak strain parametric maps. Time to peak strain has been used to illustrate synchronous contraction of different myocardial segments. Mechanical dispersion is defined as the SD of time to peak strain in all myocardial segments, reflecting contraction inhomogeneity. Such a dispersion in time to peak strain has been shown to not only correlate with the degree of myocardial fibrosis but also to be an independent predictor of ventricular arrhythmia.^12^ Myocardial segments with impaired contractility (such as after myocardial infarction) are expected to have reduced regional strain values along with corresponding delayed time to peak strain.^12^ A paradoxical *early* time to peak strain in segments with relatively *impaired* peak longitudinal strain seems to be unique, and there are no data in the literature describing this phenomenon. A possible explanation of such a paradox is altered myocardial mechanics in patients with AHCM leading to early passive deformation of distal and apical myocardial segments due to effects from basal to midmyocardial segments with less fibrosis.

However, in our case, time to peak strain has a striking drop in value as we transition from base to apex as opposed to peak systolic strain values that had a modest drop. This drop or gradient of strain values reflects on the bull’s-eye plot color mapping as a shift from true red to pink (for modest gradients) or from true red to blue (for steeper gradients). Previous reports,^1^, ^10^, ^11^ however, had patients with steeper peak systolic strain gradients displaying as a true blueberry-on-top pattern on the bull’s-eye plot of the peak systolic strain. The abovementioned reports did not include time to peak strain plots. This, in large part, is the novelty of our report. Additionally, the presence of an apical aneurysm can result in steeper peak systolic strain gradients due to significant systolic dyskinesis of the apex.^10^ The final appearance of the strain bull’s-eye plot therefore may vary from patient to patient and among different echocardiographic vendors. The recognition of such variability is important for accurate interpretation of the blueberry-on-top phenomenon in clinical practice. It is important to recognize that the blueberry-on-top strain pattern is not specific for AHCM since patients with ischemic heart disease and apical aneurysm/dyskinesia can have a similar strain pattern. Therefore, the blueberry-on-top strain pattern could be utilized as a suggestive feature of AHCM in the presence of appropriate diagnostic background for such a diagnosis, including increased regional/apical LV wall thickness and typical ECG changes. The incidence of the blueberry-on-top phenomenon by peak systolic strain or by time to peak strain has not been previously reported, and specific information about its predictive value is lacking. Yet the recognition of this finding may be helpful in raising the suspicion for AHCM in the presence of otherwise ambiguous TTE findings.

Management of HCM involves shared decision-making conversations and referral to multidisciplinary HCM centers with high levels of expertise. Moreover, cardiac imaging has a pivotal role in the management of patients with HCM, including the decision for ICD placement. There are several imaging criteria for ICD placement, which are most reliably characterized by CMR. Imaging guidelines recognize the incremental value and advantages of CMR over echocardiography for more comprehensive evaluation of patients with HCM. In patients with HCM, SCD risk assessment is recommended at initial evaluation and every 1 to 2 years thereafter, and this should include cardiac imaging to follow up on maximal LV wall thickness, LVEF, and LV apical aneurysm (class I recommendation). Moreover, all asymptomatic, phenotype-negative first-degree relatives deemed to be at risk for developing HCM based on family history or genotype status are recommended to have an initial screening with ECG and echocardiography at the time HCM is diagnosed in another family member and every 3 to 5 years thereafter for adults (the frequency of repeat testing is even shorter in children and adolescents especially in genotype-positive families and families with early-onset disease).

Cardiac magnetic resonance imaging was nondiagnostic especially at the cardiac apex due to severe ferromagnetic artifacts from BiV-PPM. In our case, the patient was unable to elevate their left arm, and there was severe ferromagnetic artifact despite using spoiled gradient echo sequence and 1.5 T magnetic field strength.

Apical HCM was initially thought to not carry additional mortality risk when compared to the general population, but recent data suggest annual cardiac death rates appear to mimic those seen in classic HCM, with rates ranging from 0.5% to 4%.^13^^,^^14^ Mortality is increased in women; this phenomenon is attributed to AF burden and pulmonary hypertension.^13^ As such, identification of alternative strain patterns may provide important insight and mortality benefits through early recognition and management of AHCM.

## Conclusion

The blueberry-on-top strain pattern is a characteristic finding in patients with AHCM and could be utilized as a supportive diagnostic finding.

## Ethics Statement

The authors declare that the work described has been carried out in accordance with The Code of Ethics of the World Medical Association (Declaration of Helsinki) for experiments involving humans.

## Consent Statement

The authors declare that since this was a noninterventional, retrospective, observational study utilizing de-identified data, informed consent was not required from the patient under IRB exemption status.

## Funding Statement

The authors declare that this report did not receive any specific grant from funding agencies in the public, commercial, or not-for-profit sectors.

## Disclosure Statement

The authors report no conflict of interest.

## Supplementary Data

Video 1 Two-dimensional TTE, apical 4-chamber view, demonstrates the massively dilated atria, small LV cavity with progressive mid-to-apical LV wall thickness, and systolic cavity obliteration.

Video 2 Two-dimensional TTE, apical 3-chamber zoomed view, demonstrates LV apical hypertrophy with an ace of spades–shaped LV cavity and systolic obliteration.

Video 3 Two-dimensional TTE, apical 5-chamber view with color-flow Doppler, demonstrates the slit-like LV cavity near the cardiac apex and mild mitral regurgitation.

Video 4 Two-dimensional TTE, apical 4-chamber zoomed view with color-flow Doppler (low Nyquist limit of ±30.8 cm/sec) demonstrates slit-like LV cavity toward the cardiac apex with marked trabeculations. Tricuspid regurgitation is also noted.

Video 5 Two-dimensional TTE, apical long-axis view after administration of an ultrasound-enhancing agent, demonstrates the slit-like LV cavity extending into the apical tip with marked trabeculations.

Video 6 Two-dimensional TTE, apical 4-chamber view after administration of an ultrasound-enhancing agent, demonstrates the slit-like LV cavity extending into the apical tip with marked trabeculations and the absence of an apical aneurysm.
